# Sustained Inhibition of VEGF and TNF-α Achieves Multi-Ocular Protection and Prevents Formation of Blood Vessels after Severe Ocular Trauma

**DOI:** 10.3390/pharmaceutics15082059

**Published:** 2023-07-31

**Authors:** Chengxin Zhou, Fengyang Lei, Jyoti Sharma, Pui-Chuen Hui, Natalie Wolkow, Claes H. Dohlman, Demetrios G. Vavvas, James Chodosh, Eleftherios I. Paschalis

**Affiliations:** 1 Department of Ophthalmology, Harvard Medical School, Boston, MA 02115, USA; gevrrod@gmail.com (C.Z.); dfylei@icloud.com (F.L.); wallace.pc.hui@gmail.com (P.-C.H.); natalie_wolkow@meei.harvard.edu (N.W.); claes_dohlman@meei.harvard.edu (C.H.D.); demetrios_vavvas@meei.harvard.edu (D.G.V.);; 2 Boston Keratoprosthesis Laboratory, Massachusetts Eye and Ear, Boston, MA 02114, USA; 3 David G. Cogan Laboratory of Eye Pathology and Ophthalmic Plastic Surgery Service, Massachusetts Eye and Ear, Boston, MA 02114, USA; 4 Angiogenesis Laboratory, Massachusetts Eye and Ear, Boston, MA 02114, USA; 5 Disruptive Technology Laboratory, Massachusetts Eye and Ear, Boston, MA 02114, USA; 6 Department of Ophthalmology and Visual Sciences, University of New Mexico School of Medicine, Albuquerque, NM 87108, USA

**Keywords:** cornea, retina, injury, biologics, VEFG, TNF, burn, degeneration, neovascularization

## Abstract

Purpose: This study aimed to develop a clinically feasible and practical therapy for multi-ocular protection following ocular injury by using a thermosensitive drug delivery system (DDS) for sustained delivery of TNF-α and VEGF inhibitors to the eye. Methods: A thermosensitive, biodegradable hydrogel DDS (PLGA-PEG-PLGA triblock polymer) loaded with 0.7 mg of adalimumab and 1.4 mg of aflibercept was injected subconjunctivally into Dutch-belted pigmented rabbits after corneal alkali injury. Control rabbits received 2 mg of IgG-loaded DDS or 1.4 mg of aflibercept-loaded DDS. Animals were followed for 3 months and assessed for tolerability and prevention of corneal neovascularization (NV), improvement of corneal re-epithelialization, inhibition of retinal ganglion cell (RGC) and optic nerve axon loss, and inhibition of immune cell infiltration into the cornea. Drug-release kinetics was assessed in vivo using an aqueous humor protein analysis. Results: A single subconjunctival administration of dual anti-TNF-α/anti-VEGF DDS achieved a sustained 3-month delivery of antibodies to the anterior chamber, iris, ciliary body, and retina. Administration after corneal alkali burn suppressed CD45^+^ immune cell infiltration into the cornea, completely inhibited cornea NV for 3 months, accelerated corneal re-epithelialization and wound healing, and prevented RGC and optic nerve axon loss at 3 months. In contrast, anti-VEGF alone or IgG DDS treatment led to persistent corneal epithelial defect (combined: <1%; anti-VEGF: 15%; IgG: 10%, of cornea area), increased infiltration of CD45^+^ immune cells into the cornea (combined: 28 ± 20; anti-VEGF: 730 ± 178; anti-IgG: 360 ± 186, cells/section), and significant loss of RGCs (combined: 2.7%; anti-VEGF: 63%; IgG: 45%) and optic nerve axons at 3 months. The aqueous humor protein analysis showed first-order release kinetics without adverse effects at the injection site. Conclusions: Concomitant inhibition of TNF-α and VEGF prevents corneal neovascularization and ameliorates subsequent irreversible damage to the retina and optic nerve after severe ocular injury. A single subconjunctival administration of this therapy, using a biodegradable, slow-release thermosensitive DDS, achieved the sustained elution of therapeutic levels of antibodies to all ocular tissues for 3 months. This therapeutic approach has the potential to dramatically improve the outcomes of severe ocular injuries in patients and improve the therapeutic outcomes in patients with retinal vascular diseases.

## 1. Introduction

Ocular injuries are often bilateral and can result in irreversible vision loss not only due to extensive corneal neovascularization (NV), conjunctivalization, and stromal scaring [1,2] but also due to intraocular complications, such as peripheral anterior synechiae (PAS), proliferative vitreoretinopathy (PVR) [3], and secondary or inflammatory glaucoma, with the latter a frequent and devastating long-term complication [4,5]. New therapies addressing damage to the anterior and posterior segment from ocular injuries are urgently needed to improve patient outcomes. Such therapies could improve not only the post-operative care but also the outcomes of subsequent penetrating keratoplasty, given that pre-existing corneal neovascularization adversely affects graft survival. Moreover, such therapies could also ameliorate complications associated with ocular injury, such as PAS, CNV, and secondary glaucoma, which are common in patients and known to gradually lead to permanent vision loss [6].

To this end, VEGF inhibitors, such as monoclonal anti-VEGF antibodies, have been proposed as an alternative to corticosteroids for the prevention and treatment of corneal neovascularization [7,8] in pre-clinical [9,10,11] and clinical studies [12,13,14]. Likewise, TNF-α inhibitors [7], including adalimumab and infliximab, were shown to provide significant neuroretinal protection against post-injury neuroinflammation in animal studies [8,15]. Moreover, combination therapy with TNF-α and VEGF inhibitors was shown to improve clinical outcomes in patients with age-related macular degeneration (AMD) and macular edema [16,17]. Thus, inhibition of angiogenesis and inflammation using neutralizing antibodies could prove to be an important therapeutic modality for pan-ocular protection after eye trauma.

Antibody delivery to ocular tissues is a challenging task compared to other tissue targets, and it may lead to significant adverse events [18]. For example, systemic administration of antibodies, especially VEGF inhibitors, only offers limited drug availability in the ocular tissue, while it exposes the whole body to the agent and can lead to major complications. In an effort to minimize potential adverse effects, topical administration of antibodies in the form of eye drops has been attempted but was not shown to achieve adequate bioavailability in the eye [19,20]. Moreover, one study has shown that prolonged topical therapy with VEGF inhibitors can lead to epithelial toxicity [13,21]. The intravitreal administration of antibodies, on the other hand, achieves excellent bioavailability in the eye but has a limited therapeutic effect on the anterior eye and cornea and is associated with rare but devastating complications, such as endophthalmitis and retinal detachment. The subconjunctival administration of antibodies is an alternative option that allows for good drug bioavailability for both the anterior segment and posterior segment [22,23]. However, antibodies undergo rapid diffusion in the subconjunctival compartment [22] compared to other competing drug elimination routes, thereby limiting the duration of the effect.

Various drug delivery systems (DDSs) have been developed to improve the stability of the sensitive protein in vivo and provide targeted, sustained protein administration [24]. To this end, we developed a novel DDS based on thermosensitive biodegradable PLGA-PEG-PLGA triblock hydrogel for extended protein delivery and further explored the combined therapeutic efficacy of anti-VEGF and anti-TNF-α given together via a DDS through a single subconjunctival injection. We demonstrated sustained antibody delivery to the cornea, iris, and retina for 3 months, without local adverse events. Furthermore, using a well-established alkali injury model, we demonstrated almost complete protection from post-injury corneal neovascularization, as well as secondary retinal and optic nerve damage. Our data suggest that sustained delivery of TNF-α and VEGF inhibitors in combination in the appropriate DDS could greatly ameliorate downstream damage from eye injuries. By using a thermosensitive, biodegradable PLGA-PEG-PLGA DDS, we allowed for the injection of the therapy though a fine needle, reduced the risk of antibody denaturization, eliminated possible adverse effects from the use of catalysts for cross-linking, and allowed for the re-administration of the therapy, as needed.

## 2. Methods

### 2.1. Thermosensitive Polymer Solution Preparation

To generate a thermosensitive, injectable, biodegradable hydrogel for the delivery of therapeutic biologics, a poly(lactide-co-glycolide)-*b*-poly(ethylene glycol)-*b*-poly (lactide-co-glycolide) triblock copolymer was dissolved (15:1 lactic acid: glycolic acid, 1750–1500–1750 Da) (AK141, PolysciTech, West Lafayette, IN, USA) in sterile water (20% *w*/*v*) at 4 °C overnight, with gentle stirring, as suggested by the manufacturer’s user guide [25]. The polymer solution was then sterilized with 30 min ultraviolet light radiation in a biosafety hood. The sterile polymer solution was stored in syringes at −20 °C until use.

To prepare the drug-loaded polymer solution, infliximab lyophilized powder (Janssen biotech, Raritan, NJ, USA) was weighed and dissolved in the sterile polymer solution at 4 °C, which is the storage temperature for the antibodies. The aflibercept injection solution (Regeneron, Tarrytown, NY, USA) was added to the solution at 4 °C. The final product contained 2 mg of therapeutic proteins (1:2 infliximab:aflibercept) in every 300 μL of polymer solution (refilled in a 1 mL Luer-Lok syringe (309628, BD, San Jose, CA, USA).

### 2.2. Rheology Test

The rheological properties of the 20% triblock polymer were measured with dynamic mechanical analyzer (Discovery HR-3, TA instruments, New Castle, DE, USA) with a 20 mm 5-degree cone. The test was performed by oscillating at an angular frequency constant 6.283 rad/s, 0.1% strain, in increments of 1 °C, ranging from 4 to 45 °C, with 1 min of temperature equilibration at each temperature, as suggested by the manufacturer.

### 2.3. Corneal Neovascularization Model

A corneal alkali injury was used to generate corneal NV, as previously published [7,8,15,18,26,27,28,29]. Rabbits were treated in accordance with the Association for Research in Vision and Ophthalmology Statement on the Use of Animals in Ophthalmic and Vision Research and the National Institutes of Health Guide for the Care and Use of Laboratory Animals. The protocol was approved by the Animal Care Committee of the Massachusetts Eye and Ear (ACC 10-033A). Dutch-Belted rabbits (*n* = 10, female, 2–2.5 kg) purchased from Envigo, Dedham, MA, USA, received an intramuscular injection of ketamine hydrochloride INJ, USP (35 mg/kg; KetaVed, VEDCO, St. Joseph, MO, USA) and xylazine (5 mg/kg; AnaSed, LLOYD, Shenandoah, IA, USA) prior to surgery. Then, 0.5% proparacaine hydrochloride (Bausch & Lomb, Tampa, FL, USA) was used for topical anesthesia to the eye before commencing alkali burn, using an 8 mm diameter filter paper soaked in 2N NaOH, applied to the center of the cornea for 20 s. After the burn, the eye was irrigated with normal saline for 15 min, as described also in prior published papers by our group (refs. [7,18,26]).

### 2.4. Subconjunctival DDS Placement

Each 20% triblock polymer was UV-radiated for 30 min on ice and then mixed with either a predetermined amount of infliximab dry powder, aflibercept stock solution, or both. The drug-laden triblock polymer solution was distributed in 1 mL syringes for in vivo injection. Each syringe was prefilled with 300 μL of drug-laden DDS and was stored at 4 °C until animal injection in vivo.

For DDS administration, chilled DDS (300 μL/syringe/injection) loaded either with 2 mg of infliximab/aflibercept antibodies (1:2) (*n* = 3), 1.3 mg of aflibercept (*n* = 3), or 2 mg of human IgG isotype (*n* = 4) (I4506-100 MG, Millipore-Sigma, Saint Louis, MO, USA) were injected at the superior bulbar subconjunctiva of eyes immediately after the post-alkali exposure irrigation, i.e., 15 min after the injury. Erythromycin ophthalmic ointment (0.5%, Bausch & Lomb, Tampa, FL, USA) was administered topically twice a day for 1 week.

### 2.5. Evaluation of Corneal NV and Epithelial Defects

An evaluation of the corneal NV was performed under general anesthesia every week for the first month and every two weeks thereafter for two additional months. Eyes were photographed with a digital SLR camera (Nikon, Melville, NY, USA) attached to a surgical microscope (S21; Carl Zeiss, Jena, Germany). A portable slit-lamp (Keeler 3010-P-2001; Keeler Americas, Malvern, PA, USA) with a cobalt blue filter was used to assess corneal epithelial defects with fluorescein staining at 10× magnification. ImageJ 1.50e software (http://imagej.nih.gov/ij/ (accessed on 28 August 2021); National Institutes of Health [NIH], Bethesda, MD, USA) was used to analyze the images. Epithelial defect was quantified as the percentage (%) of the total corneal area stained with fluorescein (pixel^2^) normalized to the whole cornea area (pixel^2^) [18,29]. Corneal NV was quantified separately for the superior and inferior cornea as the vascularized area in each half normalized to half of the corneal area (%). Illustration graphs of all the representative biomicroscopic images of corneal neovascularization and epithelial defects were manually sketched in Adobe Illustrator 2020 software (Adobe, San Jose, CA, USA) by delineating the corneal vessels and defects with a ‘pencil’ tool on top of the original photos.

### 2.6. Aqueous Humor Collection and ELISA Assay

The rabbit’s aqueous humor (AH) was collected when the animal was under general anesthesia at prespecified time points. Tropicamide ophthalmic solution (USP 1%, Akorn, Gurnee, IL, USA) and topical anesthetic (0.5% proparacaine hydrochloride, Bausch & Lomb, Tampa, FL, USA) were applied to the eye. A 30G needle combined with a 1 mL syringe was used to aspirate approximately 50~70 μL aqueous humor from the treated eye and the contralateral eye. Antibiotics eye drops were given to the eye following the procedure. Aqueous humor samples were stored in a −80 °C freezer until processed with ELISA assay. All AH samples were diluted in PBS at 1:20 and stored on ice. Human IgG sandwich ELISA assay (RAB0001, Sigma, Saint Louis, MO, USA) was performed per manufacturer’s protocol. Serially diluted human IgG standards and blank samples were analyzed along with the test samples. A standard curve was generated to calculate the human IgG concentrations in the AH. Human IgG levels were quantified in the AH of rabbits injected with IgG- and antibody-loaded DDS.

### 2.7. Histological and Immunohistochemical Evaluation of the Cornea

Rabbits were euthanized at 3 months, using intravenous Beuthanasia-D (sodium pentobarbital and phenytoin sodium, 100 mg/kg; Merck Animal Health, Madison, NJ, USA). Eyes were dissected and fixed in 4% paraformaldehyde (PFA). One half of the globes was embedded in optimal compound temperature (OCT, Sakura Fineteck, Torrance, CA, USA) for frozen tissue sections (10 µm thickness), using a cryostat (CM1950; Leica Biosystems, Buffalo Grove, IL, USA). The other half was embedded in methacrylate for tissue sections for histologic evaluation with hematoxylin and eosin (H&E) staining. Immunohistochemical assays were performed as previously described [26]. Primary antibodies were diluted in 1% BSA and incubated with tissue sections overnight at 4 °C. A secondary antibody was then applied and incubated at room temperature for two hours. Leukocyte recruitment into the tissue was evaluated with anti-CD45 antibody staining (1:100, SC-70690, mouse anti-rabbit, Santa Cruz, Dallas, TX, USA) and donkey anti-mouse secondary antibody (1:200, ab150110, Abcam, Waltham, MA, USA). Residual human IgG in the DDS and in the ocular tissues was determined using goat anti-human IgG secondary antibodyAF546 (1:200, A-21089, Thermo Scientific, Waltham, MA, USA) after blocking with 1% BSA. The total number and area of cells expressing CD45 in corneal sections were determined using the “Analyze Particles” tool in ImageJ 1.50e software, as previously described [18]. The mean ± standard deviation (SD) of different rabbits was reported.

### 2.8. Statistical Analysis

All experiments were performed with at least 3 technical replicates. Quantitative results were presented as means ± standard deviations. The normality of data was assessed by the Shapiro–Wilk test. One-way and two-way ANOVA tests were performed when data contained multiple variables and were corrected with Tukey’s. In addition to parametric tests, we confirmed results by using a non-parametric Mann–Whitney (*t*-test) and Kruskal–Wallis (ANOVA) tests. A mixed ANOVA was performed when data contained dependent variables (e.g., CNV and corneal defect area). The fixed variables were the time and treatment. Analyses were performed using the Statistical Package of Social Sciences (SPSS, IBM, Armonk, NY, USA), R Studio (Boston, MA, USA), and GraphPad Prism software Version 17 (San Diego, CA, USA). Linear and second-order polynomial functions were generated in GraphPad Prism Version 6.0 (GraphPad, La Jolla, CA, USA).

## 3. Results

### 3.1. DDS Sol–Gel Transition and Drug Release Assessment In Vitro Using Fluorescein-Conjugated Dextran

**Figure 1:**
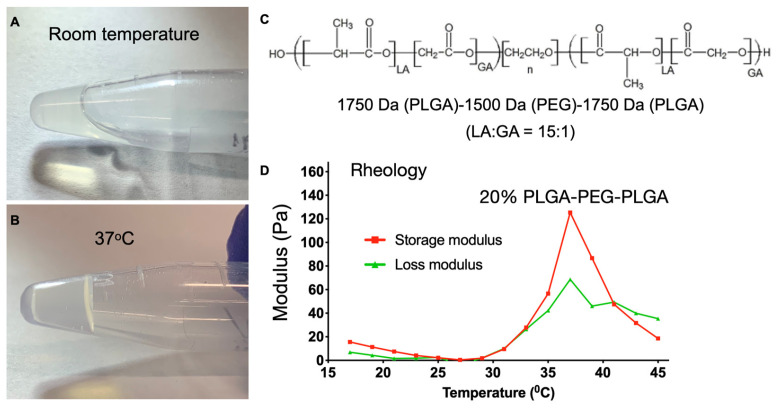
Characterization of 20% PLGA-PEG-PLGA triblock polymer. (**A**,**B**) Sol–gel transition from room temperature to 37 °C. Note that the liquid polymer becomes a gel. (**C**) Molecular structure of the triblock polymer and (**D**) rheological assessment of the triblock polymer showing sol–gel transition peak at 37 °C; *n* = 3.

The PLGA-PEG-PLGA triblock copolymer used in this study presented a sol–gel transition at ~37 °C (Figure 1A,B,D) within a few minutes. Cured 20% triblock hydrogel remains a transparent viscus at 37 °C. This sol–gel transition temperature is below a rabbit’s body temperature (~38 °C), thus allowing for the rapid formation of hydrogel-based drug reservoir upon injection, providing localized, long-term sustained release in the injection site. The 20% triblock hydrogel exhibited high water content, low stiffness, and plasticity, which can be particularly beneficial for ocular applications.

### 3.2. Long-Term Sustained Release of Antibodies into the Eye after Single Subconjunctival Injection of the DDS

**Figure 2:**
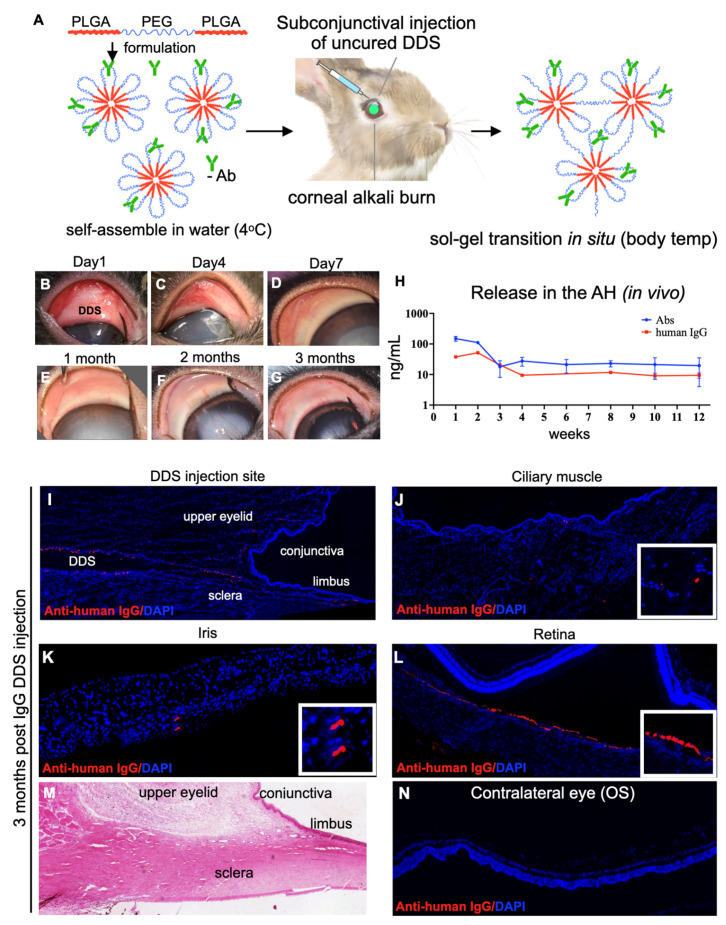
In vivo evaluation of drug release following subconjunctival (superior bulbar) administration of the 20% triblock thermosensitive drug delivery system (DDS). (**A**) Schematic representation of the DDS containing antibodies injected in the superior subconjunctival space after corneal alkali burn. (**B**–**G**) Biomicroscopic images of the upper conjunctiva and cornea harboring the DDS. The DDS slowly degraded, as evident by the regression of upper conjunctival thickening over 3 months. (**H**) ELISA analysis of aqueous humor samples demonstrated continuous release of therapeutic levels of antibodies (Abs: anti-TNF-α and anti-VEGF) in the anterior chamber for over 3 months. Likewise, IgG-DDS-treated (isotype control) eyes demonstrated a continuous release of human IgG in the anterior chamber for over 3 months. Three months after DDS injection, (**I**) the subconjunctival tissue harboring the DDS was immunopositive for human IgG, suggesting the presence of humanized antibody in the tissue. Likewise, a humanized antibody was also found in the ciliary muscle (**J**), iris (**K**), and sub-retinal space (**L**). (**M**) H&E staining of upper eyelid hosting the DDS showed no tissue abnormalities. The DDS appeared to be degraded and cleared from the tissue at 3 months. (**N**) No human IgG was present in the contralateral un-injected eyes of animals injected with either therapeutic antibodies or isotype IgG DDS; *n* = 3.

The ability to deliver anti-VEGF/anti-TNF-α inhibitors, as well as the safety and efficacy of the thermosensitive triblock polymer, was evaluated in vivo, following a single subconjunctival injection of the DDS in rabbit eyes with corneal alkali injuries. The DDS was injected as a cold liquid polymer in the superior bulbar subconjunctival 15 min after the corneal alkali burn, using a 30G needle and a 1 mL syringe. Upon injection, the polymer rapidly gelated (Figure 2A) and formed a visible drug reservoir. The DDS biodegraded over the 3 months of follow-up (Figure 2B–G). Antibody penetration in the eye was evaluated via an aqueous humor sampling and analysis of the IgG content, using a human IgG ELISA kit (RAB0001, Sigma, Saint Louis, MO, USA). Upon DDS injection, the human IgG content in the aqueous humor of eyes injected either with therapeutic antibodies (Abs) or IgG (isotype control) exhibited a rapid increase for the first two weeks after injection (Figure 2H), followed by a gradual decrease between weeks 2 and 6 and subsequent normalization between weeks 6 and 12 (Figure 2H).

Immunofluorescent staining of human IgG in cryosectioned eyelid tissues showed that, 3 months after injection of the DDS, a substantial amount of human IgG was present in the bulbar and forniceal subconjunctival connective tissue, iris, ciliary body, and retina (Figure 2I–L). Histopathological examination 3 months after injection revealed normal-appearing ocular adnexa (Figure 2M). The immunohistochemical evaluation of human IgG presence in the retina of the contralateral un-injected eye of animals treated with IgG DDS showed an absence of human IgG (Figure 2N).

### 3.3. Anti-TNF-α/Anti-VEGF DDS Treatment Completely Suppresses Corneal Angiogenesis after Injury

**Figure 3:**
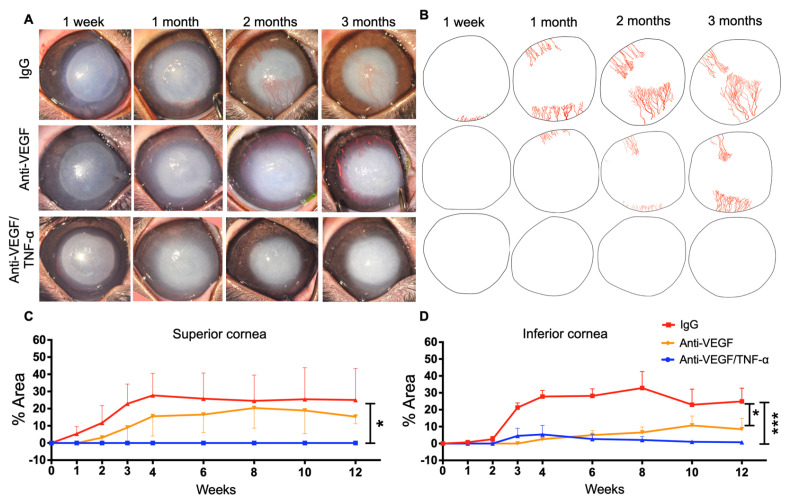
Anti-TNF-α/anti-VEGF DDS antibodies sustained released by thermogel DDS significantly halted the progression of corneal neovascularization after corneal burn injury. (**A**) Representative biomicroscopic images of burned DDS implanted rabbit eyes at specified time points. (**B**) Angiographic illustration of the corneas presented in (**A**). (**C**,**D**) CoNV area quantification in percentages of superior and inferior cornea areas, respectively. (**A**,**D**) Three months after injury, anti-TNF-α/anti-VEGF DDS conferred nearly complete blockade of CoNV ((**C**,**D**); blue curve). In contrast, eyes administrated with isotype IgG DDS exhibited extensive CoNV over 3 months (CoNV area at endpoint: ~30% of superior area; 30% of inferior cornea) ((**C**,**D**); red curves). Eyes treated with aflibercept DDS showed milder yet progressive CoNV when compared to the IgG DDS eyes during the 3-month follow-up (CoNV area at endpoint: ~15% of superior cornea; ~8% of inferior cornea) ((**C**,**D**); orange curves). Mixed ANOVA; * *p* < 0.05; *** *p* < 0.001; *n* = 3.

A central corneal alkali burn in rabbit eyes led to progressive corneal NV in the superior and inferior corneal regions of IgG-DDS-treated eyes (Figure 3A). Corneal NV reached its peak at 1.5 months post injury (25% of superior cornea; 30% of inferior cornea) (Figure 3A–D). In comparison, anti-VEGF DDS treatment led to the partial suppression of NV at 3 months, especially in the inferior cornea (maximal inferior NV: 30% with IgG vs. 8% with anti-VEGF; * *p* < 0.05; Mixed ANOVA test with Tukey’s correction) but did not completely halt progression, as the area of NV continued to grow in all rabbits during the 3 months of follow-up (Figure 3A–D). Moreover, the anti-VEGF DDS treatment showed marginal inhibition of superior corneal NV, as compared to the IgG control group (*p* > 0.05; mixed ANOVA test with Tukey’s correction). In contrast, anti-TNF-α/anti-VEGF DDS treatment led to the complete inhibition of NV in the superior and inferior cornea for 3 months, as compared to the IgG DDS treatment group (* *p* < 0.05; *** *p* < 0.001; mixed ANOVA test with Tukey’s correction; Figure 3A–D).

### 3.4. Anti-TNF-α/Anti-VEGF DDS Treatment Improves Corneal Epithelial Healing after Injury

**Figure 4:**
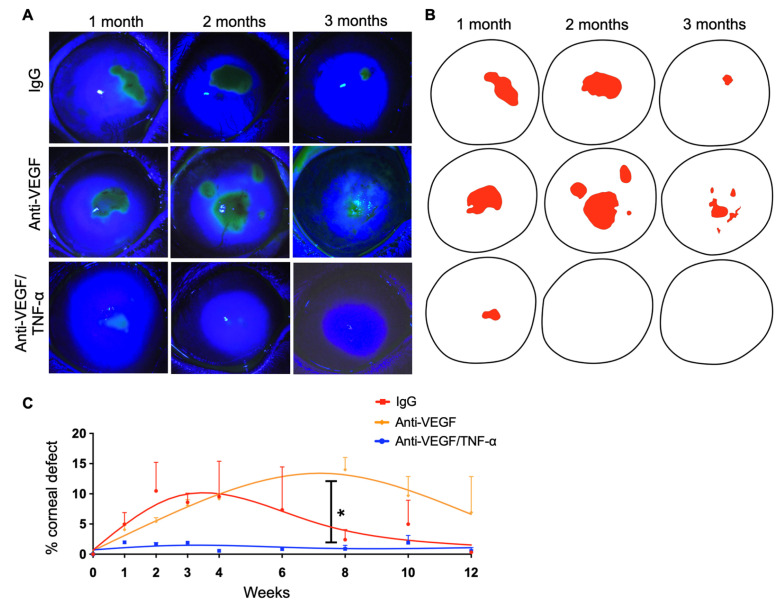
Anti-TNF-α/anti-VEGF DDS treatment reduced corneal epithelial defect in the injured cornea. (**A**) Slit-lamp biomicroscopy of fluorescein-stained rabbit eyes implanted with different DDSs. (**B**) Image reconstruction of corneal epithelial defects in the burned DDS-implanted corneas shown in (**A**). (**C**) Corneal-epithelial-defect quantification in percentage of total corneal area. Anti-TNF-α/anti-VEGF DDS treatment significantly suppressed the development of epithelial defect on the burned cornea, as compared to the aflibercept group and IgG groups; * *p* < 0.05; mixed ANOVA test with Tukey’s multiple comparison test; *n* = 3.

IgG-DDS-treated eyes had persistent epithelial defects even at 1 month after injury (10% of cornea area), which gradually reduced in subsequent months (Figure 4A–C). In eyes treated with anti-VEGF DDS, persistent epithelial defects peaked 2 months after injury (15% of cornea area), without resolution throughout the 3 months (Figure 4A–C). In contrast, combined anti-TNF-α/anti-VEGF DDS treatment led to reduced corneal epithelial defects (2% of cornea area) at 1 month and complete corneal re-epithelialization at 2 months, with a stably intact epithelium when measured at 3 months (Figure 4A–C). Quantification of the area of epithelial defect confirmed the effect of combination anti-TNF-α/anti-VEGF DDS as compared to the other two treatments (Figure 4C; * *p* < 0.05, mixed ANOVA test with Tukey’s multiple comparison test).

**Figure 5:**
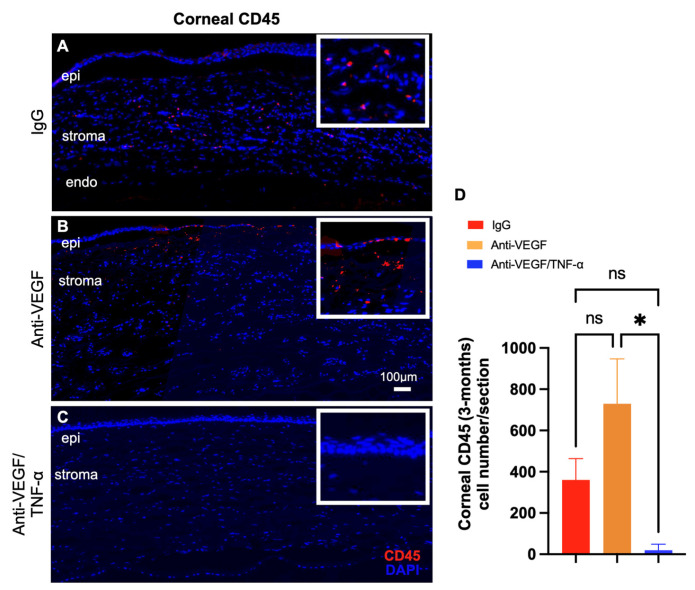
Corneal inflammation and leukocyte infiltration. (**A**) Marked CD45+ cell accumulation in the cornea of IgG-DDS-treated (**A**) or aflibercept-DDS-treated (**B**) eyes at 3 months of injury. (**C**) Remarkable reduction in CD45+ cell accumulation in the cornea following subcon-junctival injection of anti-TNF-α/anti-VEGF DDS. (**D**) Quantification of the CD45 + cell number within the corneal tissue shows statistically significant reduction in the anti-TNF-α/anti-VEGF DDS group, as compared to control IgG DDS or anti-VEGF DDS groups; * *p* < 0.05 (one-way ANOVA with Tukey’s); ns: non-significant; *n* = 3.

As shown by the immunofluorescent staining of rabbit corneas, combined anti-TNF-α/anti-VEGF DDS treatment significantly reduced CD45^+^ immune cell infiltration into the injured cornea (Figure 5C,D), as compared to IgG DDS (Figure 5A,D) or anti-VEGF DDS treatment (Figure 5B,D; * *p* < 0.05, one-way ANOVA test with Tukey’s multiple comparison test).

### 3.5. Anti-TNF-α/Anti-VEGF DDS Treatment Prevents Retinal and Optic Nerve Damage after Injury

**Figure 6:**
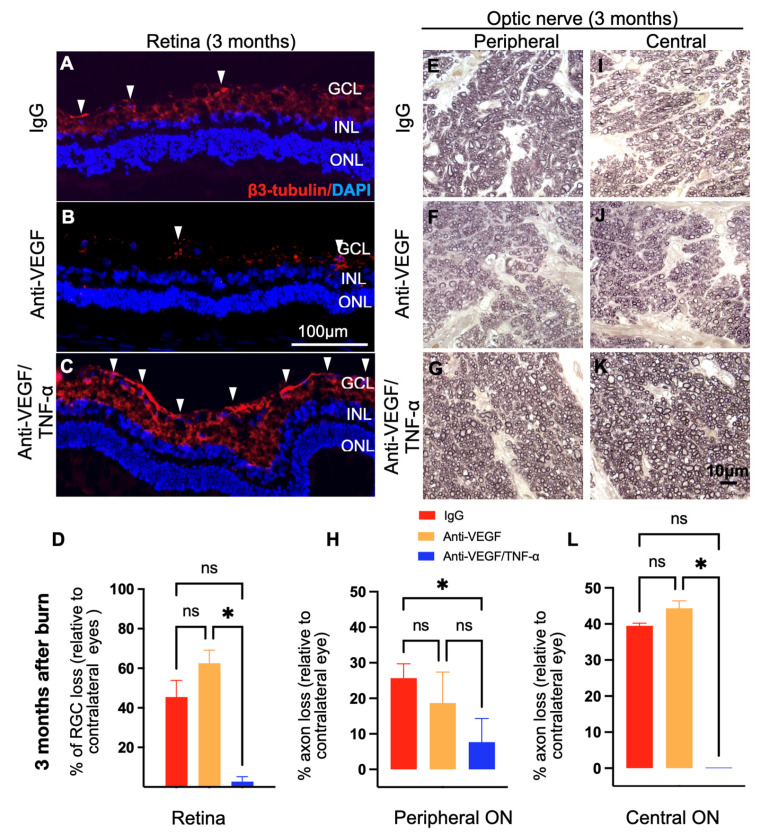
Anti-TNF-α/anti-VEGF DDS treatment effectively ameliorated retinal neuropathy and optic nerve degeneration in the injured eyes. (**A**–**D**) Three months after corneal burn, IgG- or aflibercept-DDS-treated rabbits exhibited significant retinal ganglion cell loss, as indicated by a retinal ganglion cell marker β3-tubulin (red color). In contrast, the anti-TNF-α/anti-VEGF DDS provided almost complete protection against RGC loss. Arrow head: a normal ganglion cell. (**E**–**G**) Representative PPD staining of peripheral rabbit optic nerves (63× obj). (**H**) Loss of normal nerve axon in peripheral optic nerves relative to the contralateral intact optic nerve. (**I**–**K**) Representative PPD staining of central rabbit optic nerves (*63× obj*). (**L**) Loss of normal nerve axon loss in central optic nerves relative to the contralateral intact optic nerve. (**E**–**L**) The protective effects of the anti-TNF-α/anti-VEGF DDS was confirmed with PPD staining of the optic nerves, which otherwise showed marked axonal degeneration in the IgG- and aflibercept-DDS-treated eyes and almost complete retention of the nerve axons in anti-TNF-α/anti-VEGF-DDS-treated eyes. One-way ANOVA with Tukey’s correction; * *p* < 0.005, ns: non-significant; *n* = 3. GCL = ganglion cell layer. INL = inner nuclear layer. ONL = outer nuclear layer. RGC = retinal ganglion cell.

We previously demonstrated that corneal alkali injury is associated with secondary retinal and optic nerve degeneration in mice, rabbits, and humans [26] and that, at least in experimental models, this damage is mediated by secondary inflammation independently of the intraocular pressure [26]. In alkali-injured eyes, combined anti-TNF-α/anti-VEGF DDS treatment significantly reduced post-injury retinal ganglion cell loss at 3 months, as compared to the IgG DDS treatment (2.7% RGC loss vs. 45% RGC loss, respectively; *p* < 0.01; Figure 6A–D). RGC loss was similar between the anti-VEGF- and IgG-DDS-treated groups (63% RGC loss vs. 45% RGC loss, respectively; *p* > 0.05. Figure 6B–D). Combined anti-TNF-α/anti-VEGF DDS treatment also significantly reduced peripheral and central optic nerve axon loss, as compared to IgG (Figure 6E,H,I,K) and anti-VEGF DDS treatments (Figure 6E–L; * *p* < 0.05, ** *p* < 0.01, *** *p* < 0.001, and **** *p* < 0.0001; mixed ANOVA test with Tukey’s correction).

## 4. Discussion

In the experiments shown herein, we leveraged a tunable thermosensitive PLGA-PEG-PLGA triblock co-polymer to achieve sustained delivery of VEGF and TNF-α inhibitors to the cornea, iris, and retina for 3 months. Combined treatment with antibodies to both VEGF and TNF-α completely inhibited corneal NV and remarkably reduced retinal and optic nerve damage after corneal alkali injury. Moreover, combined treatment limited a known critical adverse effect of anti-VEGF therapy, specifically the retardation of corneal epithelialization and wound healing [13,18], as manifested by a persistent epithelial defect [30].

Previous studies in our group focused on developing a non-biodegradable macro-porous DDS for anti-TNF-α or anti-VEGF therapy to the eye [18,29,31]. These studies required placement of the DDS in the subconjunctival space, using an incision in the conjunctiva. Here, we advanced this technology and leveraged it on a biodegradable thermosensitive PLGA-PEG-PLGA triblock co-polymer that is able to administer VEGF and TNF-α inhibitors to the eye through a single subconjunctival injection with a 30G needle. The DDS is delivered in liquid phase and becomes a gel upon contact with the tissue at body temperature. Gelation requires no catalysts and does not generate toxic by-products. Our studies, performed in live animals, confirmed a first-order release kinetics for 3 months in the aqueous humor of rabbits and delivery of therapeutic levels of antibodies to the cornea, iris, and retina for 3 months. Notably, this resulted in not only the complete inhibition of corneal NV but reduced retina and optic nerve damage after alkali injury to the eye, a smoldering injury notoriously difficult to mitigate with current ophthalmic treatments [4,5,6,7,26,32]. It is not clear how long it takes for the thermosensitive DDS to completely dissolve after injection in the subconjunctival space. Our results indicate the presence of human IgG in the aqueous humor and subconjunctival space 3 months after the injection of the DDS. In contrast, no IgG was detected in the contralateral eye, suggesting that IgG entry into the eye is due to drug diffusion from the DDS rather than from the systemic circulation. Previous studies are supportive, showing a similar time of degradation in vivo of a similar polymer [33].

In contrast to single-target therapy with either VEGF or TNF-α inhibitors, the concomitant inhibition of TNF-α and VEGF, as demonstrated in this study, is therapeutically superior in preventing cornea neovascularization after alkali injury and minimizing the risk of ocular complications from anti-VEGF administration to the eye [30,34,35]. Such a therapeutic outcome may not be entirely attributed to the anti-angiogenic effect of VEGF inhibitor but rather to a synergic effect of the TNF-α inhibitor. This is supported by current data showing that aflibercept DDS (treatment control) did not achieve the same level of anti-neovascularization of the cornea and, in fact, led to a persistent epithelial defect that was not present in the combined regimen. Likewise, neither anti-VEGF DDS nor anti-TNF-α DDS therapy alone, as demonstrated in the published literature, was able to completely inhibit long-term corneal NV in rabbit corneal alkali injury [18,29]. Collectively, the data suggest that the simultaneous inhibition of TNF-α and VEGF is more effective than either therapy alone [18,36,37,38,39,40,41]. It is worth noting that the concomitant inhibition of VEGF and TNF-α was previously shown to improve clinical outcomes in AMD and macular edema patients [16,17], thus confirming our observations in this study and suggesting that the concomitant inhibition of VEGF and TNF-α could be a preferred therapy for ocular burns.

Importantly, the proposed therapy also offers significant protection to the neuroretina and optic nerve. A single administration of the anti-TNF-α/anti-VEGF DDS led to a dramatical inhibition of retinal ganglion cells and optic nerve axon loss at 3 months after ocular alkali burn, an injury known to result in severe retinal pathology in humans and animals [5,26,32,42,43]. In previous studies, we showed that TNF-α is the primary mediator of retinal and optic nerve damage [15,18,26,28,29] through the modulation of the neuroglia phenotype [8,27]. Here, we showed that the administration of a total dose of 0.7 mg of TNF-α inhibitor results in almost complete retinal and optic nerve protection for 3 months, which is an important improvement to previous studies that used high-dose systemic TNF-α inhibitors. We expect that this treatment modality can substantially reduce the risk of systemic exposure to the drug without compromising the therapeutic outcomes to the eye.

To what extend the DDS contributes to the therapeutic outcome of TNF-α/VEGF inhibition is not clear, but PLGA-PEG-PLGA DDS has been shown to extend drug bioavailability for months [44]. When TNF-α was injected into the subconjunctival space in solution, no human IgG was detected in the eye 3 months after the injection (paper in review). Here, the DDS improves the drug bioavailability of antibodies after injection. A separate study assessing the effect of the DDS in the therapeutic outcome is warranted.

In summary, this is the first study to show multi-ocular protection after alkali injury, using a single administration of a DDS that delivers TNF-α and VEGF inhibitors to the cornea, iris, and retina for 3 months. The proposed combination therapy is therapeutically superior to anti-VEGF therapy alone against post-injury corneal NV and offers improved retinal and optic nerve protection, with minimal risk of systemic exposure to the antibodies. These results may have practical clinical implications for the treatment of patients with ocular injuries, and further clinical studies are warranted.

## 5. Conclusions

The concomitant inhibition of TNF-α and VEGF using a sustained polymer drug delivery method achieved complete inhibition of corneal neovascularization for 3 months after a corneal alkali burn and prevented irreversible damage to the retina and optic nerve. This therapeutic approach has the potential to dramatically improve the outcomes of severe ocular injuries in patients and improve the treatment of retinal neovascular diseases.

## Data Availability

Not applicable.
